# Phytogeographical Implication of *Bridelia* Will. (Phyllanthaceae) Fossil Leaf from the Late Oligocene of India

**DOI:** 10.1371/journal.pone.0111140

**Published:** 2014-10-29

**Authors:** Gaurav Srivastava, R.C. Mehrotra

**Affiliations:** Birbal Sahni Institute of Palaeobotany, Lucknow, India; Agharkar Research Institute, India

## Abstract

**Background:**

The family Phyllanthaceae has a predominantly pantropical distribution. Of its several genera, *Bridelia* Willd. is of a special interest because it has disjunct equally distributed species in Africa and tropical Asia i.e. 18–20 species in Africa-Madagascar (all endemic) and 18 species in tropical Asia (some shared with Australia). On the basis of molecular phylogenetic study on *Bridelia*, it has been suggested that the genus evolved in Southeast Asia around 33±5 Ma, while speciation and migration to other parts of the world occurred at 10±2 Ma. Fossil records of *Bridelia* are equally important to support the molecular phylogenetic studies and plate tectonic models.

**Results:**

We describe a new fossil leaf of *Bridelia* from the late Oligocene (Chattian, 28.4–23 Ma) sediments of Assam, India. The detailed venation pattern of the fossil suggests its affinities with the extant *B. ovata, B. retusa* and *B. stipularis*. Based on the present fossil evidence and the known fossil records of *Bridelia* from the Tertiary sediments of Nepal and India, we infer that the genus evolved in India during the late Oligocene (Chattian, 28.4–23 Ma) and speciation occurred during the Miocene. The stem lineage of the genus migrated to Africa via “Iranian route” and again speciosed in Africa-Madagascar during the late Neogene resulting in the emergence of African endemic clades. Similarly, the genus also migrated to Southeast Asia via Myanmar after the complete suturing of Indian and Eurasian plates. The emergence and speciation of the genus in Asia and Africa is the result of climate change during the Cenozoic.

**Conclusions:**

On the basis of present and known fossil records of *Bridelia*, we have concluded that the genus evolved during the late Oligocene in northeast India. During the Neogene, the genus diversified and migrated to Southeast Asia via Myanmar and Africa via “Iranian Route”.

## Introduction

**Figure 1:**
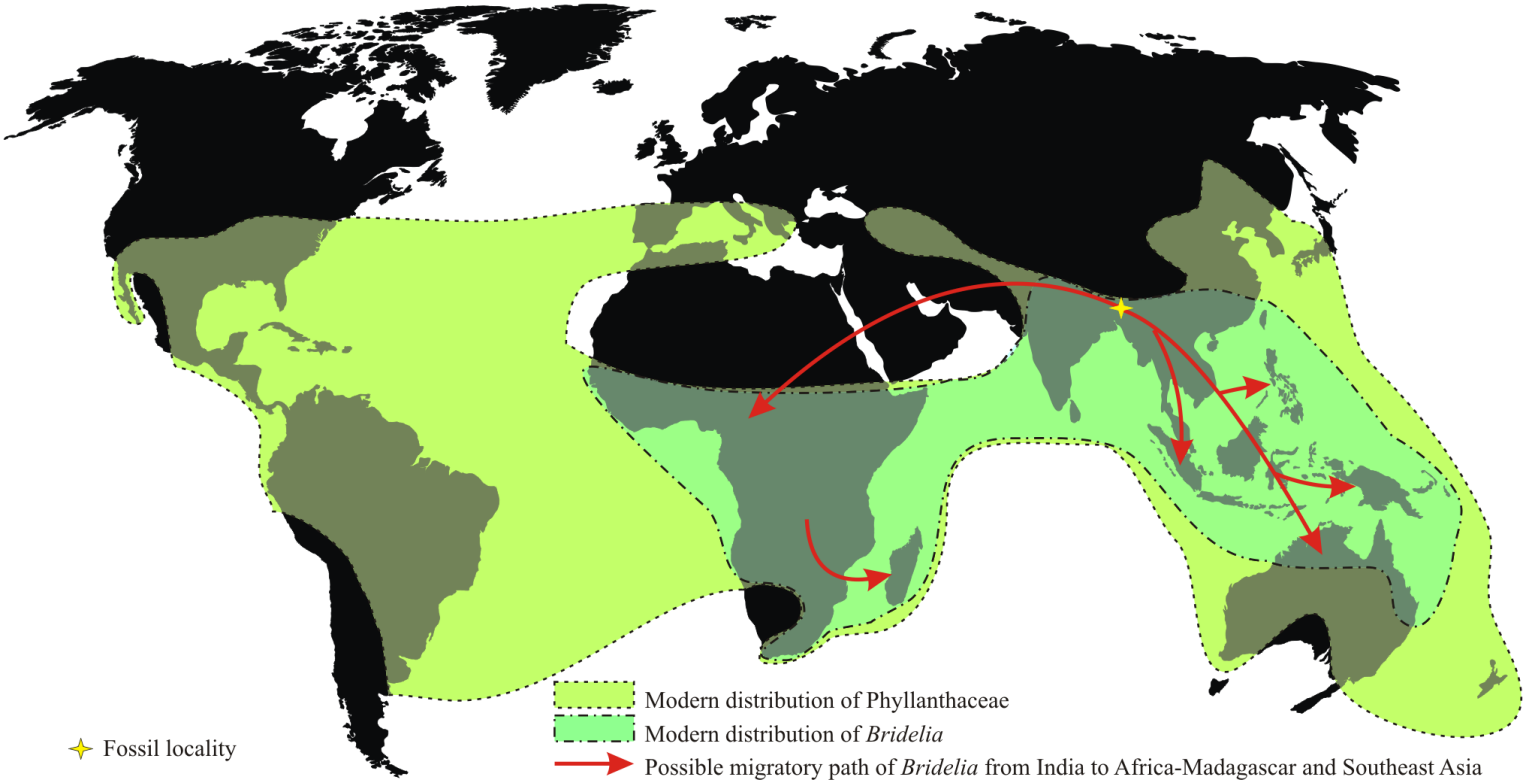
Map showing the modern distribution of the family Phyllanthaceae and *Bridelia* [1], [64].

The family Phyllanthaceae has a predominantly pantropical distribution (with a few temperate elements) [1] (Figure 1) consisting of morphologically diverse, ∼60 genera and 2000 species. The family was separated from the Euphorbiaceae *s.l.* (*sensu lato*) on the basis of molecular data [2]. The pollen evidence indicates that the family became well diversified by the Eocene [3], [4]. Of its several genera, *Bridelia* Willd. is of a special interest because it has disjunct equally distributed species in Africa and tropical Asia i.e. 18–20 species in Africa and Madagascar (all endemic) and 18 species in tropical Asia (some shared with Australia) [5]–[7]. On the basis of molecular phylogenetic study on *Bridelia*, it has been suggested that the genus evolved around 33±5 Ma (i.e. the stem age) and radiated by the 10±2 Ma (i.e. the crown group) [8]. Fossil records of such taxon are equally important to support the molecular phylogenetic studies.

**Figure 2:**
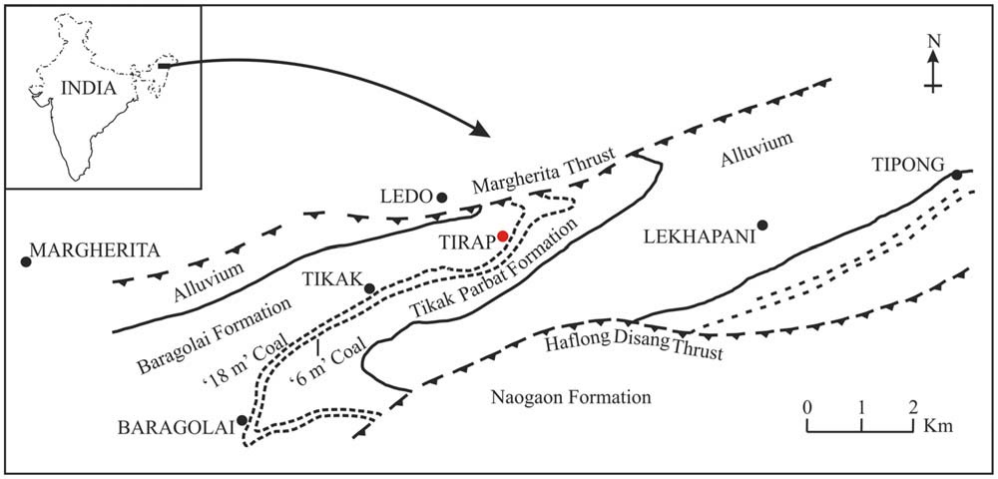
Simplified geological map of the Makum Coalfield, Assam, India showing the fossil locality (red circle) [65].

**Figure 3:**
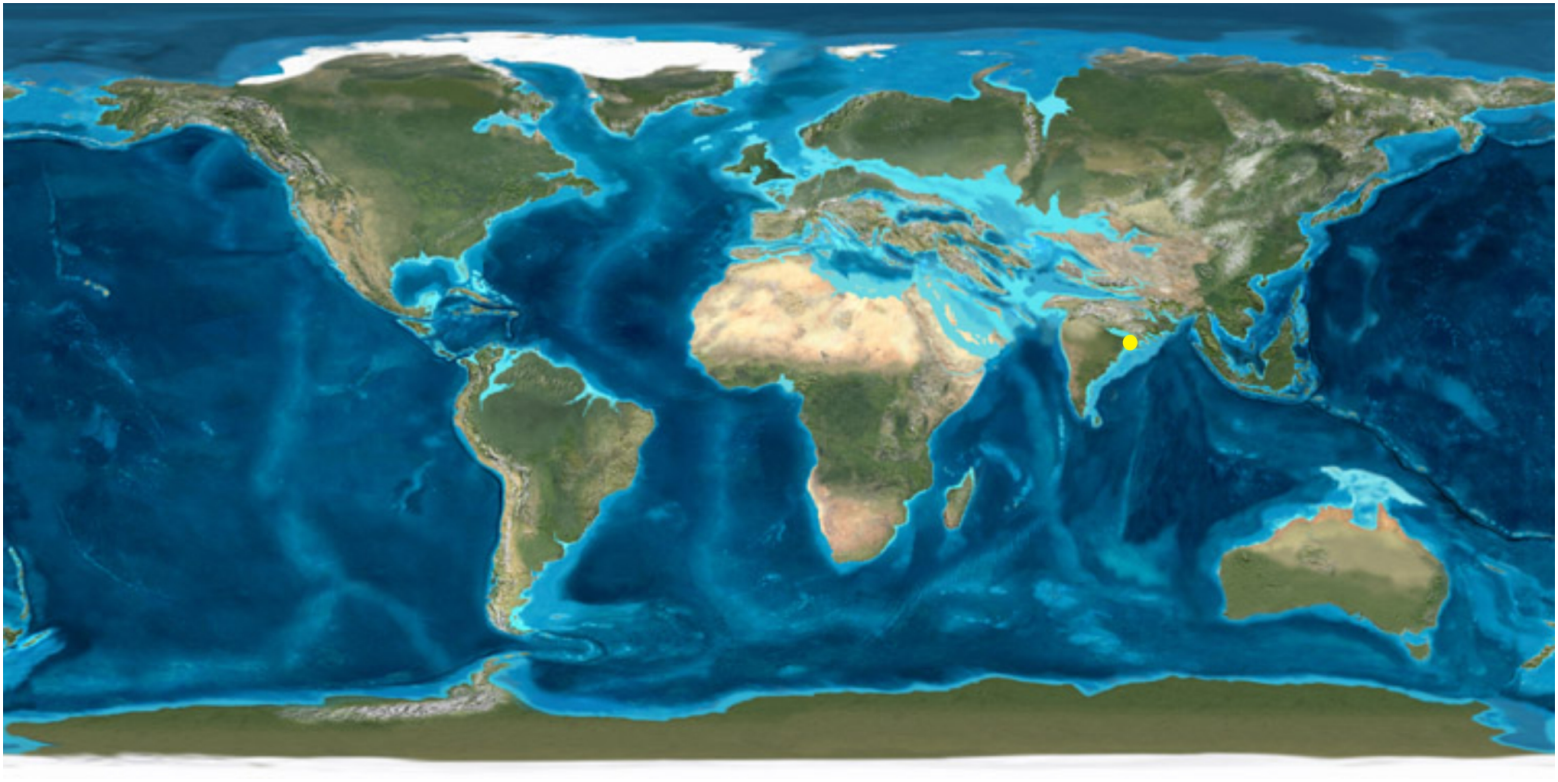
Map showing the fossil locality (yellow dot) in palaeogeographic map during the Oligocene [66].

In the present paper we describe a new leaf impression/compression of *Bridelia* from the late Oligocene (Chattian, 28.4–23 Ma; [9]) sediments of Makum Coalfield (27°15′–27° 25′ N), Assam, India (Figure 2) which was located at low palaeolatitude (i.e. 10°–15° N) during the period [10]. The suturing of the Indian plate with the Eurasian plate, during the aforesaid period, was not complete to facilitate the plant migration (Figure 3) [11]–[13]. An attempt has also been made to discuss its origin and dispersal.

### Regional geology

The Makum Coalfield is a well known basin having exposure of the late Oligocene sediments. The coalfield is important because (i) it is one of the largest coal producing basins in northeast India and (ii) it contains a well diversified low latitude palaeoflora [14]–[22]. Infact, there is no other Oligocene sedimentary basin in the Indian sub-continent which contains such a rich and diversified assemblage of fossil plants. The basin was situated at a low palaeolatitude i.e. ∼10°–15° N (Figure 3) [10] and the sediments were deposited in a deltaic, mangrove or lagoonal environment [15], [23]–[25]. The coalfield is made up of Baragolai, Ledo, Namdang, Tikak, Tipong and Tirap collieries, lies in between the latitudes 27° 15′–27° 25′ N and longitudes 95° 40′–95° 55′ E (Figure 2) and is located along the outermost flank of the Patkai range. On the southern and southeastern sides are the hills which rise abruptly to heights of 300–500 m from the alluvial plains of the Buri Dihing and Tirap rivers, respectively.

The fossils collected for the present study belong to the Tikak Parbat Formation being considered to be late Oligocene (Chattian, 28.4–23 Ma; [9]) in age on the basis of regional lithostratigraphy [26], remote sensing [27] and biostratigraphic controls [24].

The Tikak Parbat Formation constitutes alternations of sandstone, siltstone, mudstone, shale, carbonaceous shale, clay and coal seams [28]. However, the plant remains are mainly confined to the grey carbonaceous and sandy shales. The formation is underlain by 300 m of predominantly massive, micaceous or ferruginous sandstones that incorporate the Baragolai Formation, which is successively underlain by 1100–1700 m of thin-bedded fine-grained quartzitic sandstones with thin shale and sandy shale partings that constitute the Naogaon Formation [29]. Together the three formations represent the Barail Group (Figure 2). In this group, there is an upward trend of marine to non-marine palaeoenvironment which symbolizes the infilling of a linear basin on the eastern edge of the Indian plate. The detailed sedimentary information of the Tirap mine section was given by Kumar et al. [24].

## Materials and Methods

Material for the present study was collected from the Tirap colliery of the Makum Coalfield, Tinsukia District, Assam. The prior permission was taken from the General Manager, Northeastern Coalfield, Margherita, Assam, India for the collection of fossil plants. The specimen was first cleared with the help of a fine chisel and hammer and then photographed in natural low angled light using 10 megapixel digital camera (Canon SX110). The terminology used in describing the fossil leaf is based on Hickey [30], Dilcher [31] and Ellis et al. [32]. Attempts were made to extract cuticle from the leaf but it did not yield. The permission was taken from the Directors, Forest Research Institute, Dehradun and the Botanical Survey of India, Kolkata for the herbarium consultation. The fossil plant was identified with the help of herbarium sheets of the extant plant available there. The type specimen (no. BSIP 40115) is housed in the museum of the Birbal Sahni Institute of Palaeobotany, Lucknow, India.

### Nomenclature

The electronic version of this article in Portable Document Format (PDF) in a work with an ISSN or ISBN will represent a published work according to the International Code of Nomenclature for algae, fungi, and plants, and hence the new names contained in the electronic publication of a PLOS ONE article are effectively published under that Code from the electronic edition alone, so there is no longer any need to provide printed copies. The online version of this work is archived and available from the following digital repositories: PubMed Central, LOCKSS.

## Results

### Systematic description

**Order.** Malpighiales Juss. (1820) [33]

**Family.** Phyllanthaceae Martinov (1820) [34]

**Subfamily.** Phyllanthoideae Asch. (1864) [35]

**Tribe.** Bridelieae Müll. Arg. (1864) [36]

**Genus.** *Bridelia* Willd. (1806) [37]

**Species.** *B. makumensis* Srivastava and Mehrotra, sp. nov.

**Figure 4:**
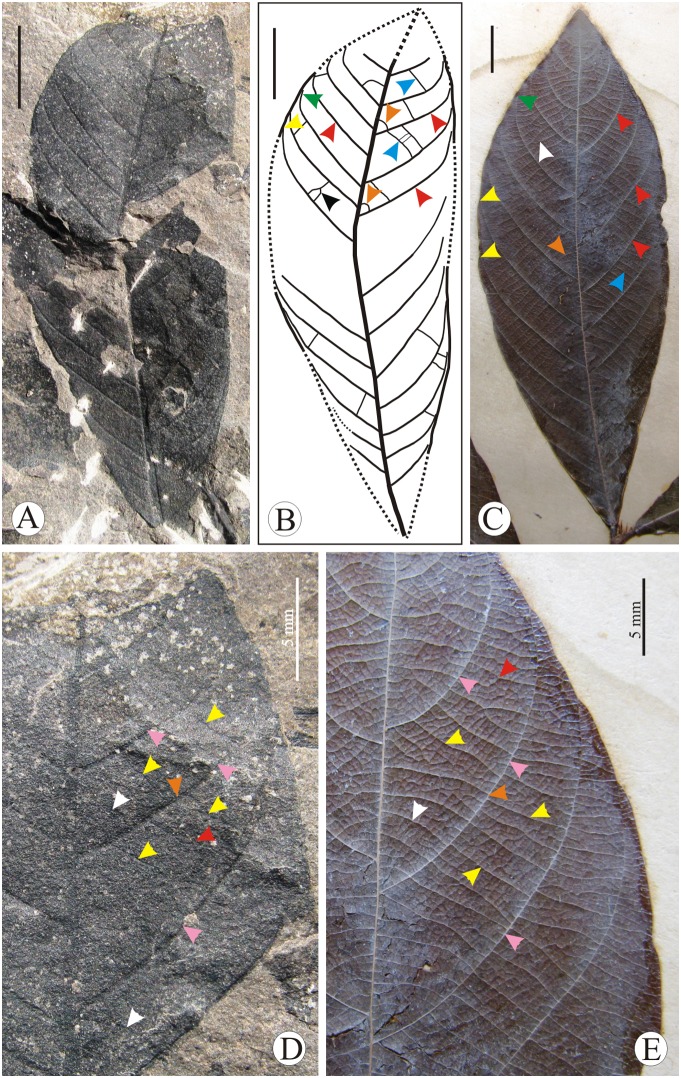
*Bridelia* leaves. A. Fossil leaf of *Bridelia makumensis* sp. nov. showing shape, size and venation pattern. B**.** Text diagram of the fossil leaf showing craspedodromous and eucamptodromous venation (yellow and green arrows), secondary veins (red arrows) and percurrent, recurved and forked tertiary veins (blue, orange and black arrows). C. Modern leaf of *Bridelia retusa* showing craspedodromous and eucamptodromous venation (yellow and green arrows), secondary veins (red arrows) and percurrent and recurved tertiary veins (blue and orange arrows). D. Enlarged portion of the fossil leaf showing secondary veins (pink arrows), percurrent, recurved and forked tertiary veins (yellow, white and red arrows); predominantly alternate tertiary veins (orange arrow). E. Modern leaf of *Bridelia retusa* showing secondary veins (pink arrows); percurrent, recurved and forked tertiary veins (yellow, white and red arrows) Scale bar = 1 cm, unless mentioned.

**Figure 5:**
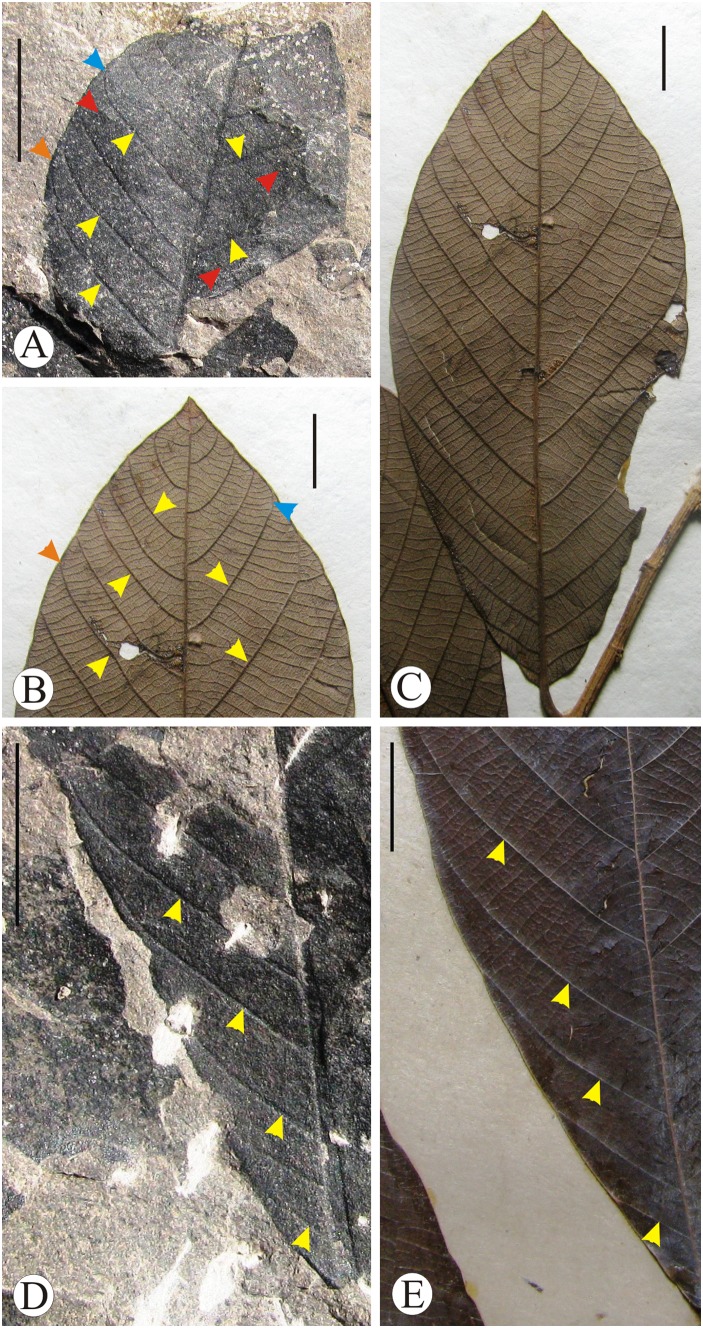
*Bridelia* leaves. A. Enlarged apical portion of the fossil leaf showing craspedodromous and eucamptodromous venation (orange and blue arrows), secondary veins (yellow arrows) and percurrent tertiary veins (red arrows). B. Apical portion of the modern leaf of *B. stipularis* showing similar craspedodromous and eucamptodromous venation (orange and blue arrows) as found in the fossil and secondary veins (yellow arrows). C. Modern leaf of *B. stipularis* showing shape, size and venation pattern. D. Basal portion of the fossil leaf showing course of secondary veins (yellow arrows). E. Basal portion of the modern leaf of *B. stipularis* showing similar course of secondary veins as found in the fossil (yellow arrows).

**Figures.** 4A, B, D; 5A, D

**Holotype.** Specimen No. BSIP 40115

**Horizon.** Tikak Parbat Formation

**Locality.** Tirap Colliery, Tinsukia District, Assam (27° 17′ 20″ N; 95° 46′ 15″ E)

**Age.** Late Oligocene (Chattian, 28.4–23 Ma)

**Number of specimens studied.** One.

#### Description

Leaf nearly complete, simple, symmetrical, microphyll, elliptic, preserved lamina length 5.2 cm (estimated lamina length 7.3 cm), maximum width near the middle portion 2.4 cm; apex not preserved but seems to be acute-obtuse; base slightly broken, asymmetrical, acute, normal; margin entire but slightly crenate seen on the distal portion, seemingly wavy; texture appearing chartaceous; attachment with petiole not preserved; venation pinnate, simple craspedodromous to eucamptodromous; primary vein stout in thickness, curved; secondary veins 11 pairs visible, 0.2–0.4 cm apart, predominantly alternate, angle of divergence moderate acute (48°–62°), smoothly and sometimes abruptly curved up near the margin, attachment with the primary vein normal, rarely decurrent; intersecondary veins absent; tertiary veins simple percurrent, recurved, forked, oblique to mid-vein, angle of origin AO, AA, AR, predominantly alternate, close; marginal ultimate venation fimbriate; quaternary veins orthogonal.

#### Affinities

The characteristic features of the fossil leaf such as elliptic shape, crenate margin, craspedodromous to eucamptodromous venation, moderate acute angle of divergence of secondary veins, percurrent to recurved tertiary veins and fimbriate marginal venation suggest its affinity with *Bridelia* of the family Phyllanthaceae. A large number of species of *Bridelia* such as *B. assamica* Hook.f., *B. cinnamomea* Hook.f., *B. glauca* Blume, *B. insulana* Hance, *B. ovata* Decne. (syn. *B. burmanica* Hook.f.), *B. retusa* (L.) A. Juss. (syn. *B. squamosa*), *B. stipularis* (L.) Blume (syn. *B. scandens*), *B. tomentosa* Blume and the species of its sister genus *Cleistanthus* Hook.f. ex Planch such as *C. collinus* (Roxb.) Benth. ex Hook.f., *C. malabaricus* Müll.Arg. and *C. monoicus* (Lour.) Müll.Arg. were studied and compared in the herbarium of the Forest Research Institute, Dehradun and the Central National Herbarium, Howrah.

In *B. assamica*, *B. cinnamomea*, *B. glauca*, *B. scandens* and *Cleistanthus monoicus* the angle of divergence of secondary veins is narrow-moderate acute which is in contrast to the present fossil. In *B. insulana*, *B. tomentosa*, *Cleistanthus collinus* and *C. malabaricus* the distance between the two secondary veins is greater than the present fossil. In the venation pattern the fossil shows maximum similarity with *B. retusa* (Figure 4C, E) and *B. stipularis* (Figure 5B, C, E) but differs from them in having asymmetrical base. In having asymmetrical base our fossil shows resemblance with *B. ovata* where base varies from asymmetrical ([38], Plate 14, Figure 2) to symmetrical (CNH Herbarium sheet no. 400497). However, angle of divergence of secondary veins is more acute in *B. ovata* than that of our fossil. It appears that our fossil shows a combination of characters found in *B. ovata*, *B. retusa* and *B. stipularis*. The comparable species are distributed throughout the hotter parts of India, along the foot of the Himalaya, south India, Malacca, Malayan Peninsula, Myanmar, Sri Lanka, Philippines and tropical Africa [39].

As far as fossil leaf records of *Bridelia* are concerned they are known mainly from Nepal and India. Two fossil species of the genus, namely *B. mioretusa* and *B. siwalica* are known from the Siwalik sediments (late Miocene) of Surai Khola, western Nepal [38]. Three more fossil species of *Bridelia*, namely *B. stipularis* and *B. verucosa* have been described from the Middle Siwalik sediments of Darjeeling, West Bengal [40], while another species viz., *B. oligocenica* is known from the late Oligocene sediments of Assam [15]. All the aforesaid fossils are different from the present fossil in a combination of characters (Table 1). Under such circumstances a new species, *B. makumensis* Srivastava and Mehrotra, sp. nov. is created and the specific epithet is after the fossil locality.

**Table 1:** Comparative chart of the known fossil leaves of *Bridelia* from the Cenozoic sediments.

		Lamina							
Fossil taxa	Modern Comparable Forms	Apex	Base	Margin	Shape	Balance	Venation pattern	2^0^ veins	3^0^ veins
*B.oligocenica* Awasthi and Mehrotra [15]	*B. retusa*	NP	Acute	Entire	Elliptic	Symmetrical	Eucamptodromous	Narrow acute	?Percurrent
*Bridelia mioretusa* Prasad and Pandey 30]	*B. retusa*	Acute	NP	Entire	Elliptic	Symmetrical	Craspedodromous- eucamptodromous	Narrow acute	Percurrent
*B. siwalika* Prasad and Pandey [30]	*B.ovata*	NP	Obtuse,asymmetrical	Entire	Obovate	Asymmetrical	Eucamptodromous	Wide acute	Percurrent
*B. stipularis* Pathak 31]	*B. stipularis*	Acute-Obtuse	Round	Entire	Elliptic	Symmetrical	Eucamptodromous	Wide acute	NG
*B. verucosa* Pathak 31]	*B. verucosa*	Obtuse	Obtuse	Entire	Elliptic	Symmetrical	Eucamptodromous	Narrow acute	NG
*B. makumensis* Srivastava and Mehrotra	*B. ovata, B. retusa* and*B. stipularis*	Seemingly acute-obtuse	Acute, asymmetrical	Entire-slightly crenate	Elliptic	Symmetrical	Craspedodromous- eucamptodromous	Moderate acute	Percurrent

NP = Not preserved;

NG = Not given.

## Discussion

### Fossil wood record of *Bridelia*

As far as the fossil records of *Bridelia* are concerned, they are known in the form of leaves and woods. The leaf fossil records have been discussed in the affinities (see section affinities). Fossil woods of *Bridelia* are known from various Tertiary sediments of central South Asia, temperate Asia, tropical Africa, Northern Africa, Europe, America and Australia [41] but their affinities with modern *Bridelia* are uncertain because of the homogeneity in wood characters of various genera of Euphorbiaceae *s.l*.

Bailey [42] instituted the genus *Paraphyllanthoxylon* for the fossil woods resembling Phyllanthoid Euphorbiaceae that includes the genus *Bridelia* also. However, he was not sure of its affinities. Wheeler et al. [43] after studying various species of *Paraphyllanthoxylon* concluded that the genus can not be assigned with certainty to the Euphorbiaceae because of its similarities to other families.

Ramanujam [44] instituted the genus *Bischofioxylon* for the fossil woods resembling *Bischofia* and described *Bischofioxylon miocenicum* from south India. Mädel [45] had suggested its affinities with *Bridelia* and merged it into another organ genus *Bridelioxylon* Mädel. Therefore, Bande [46] established *Bischofinium* for the fossil woods resembling *Bischofia*. Awasthi [47] suggested that neither *B. miocenicum* Ramanujam nor *Biscofinium* Bande belongs to *Bischofia* or *Bridelia*. The generic diagnosis of *Bridelioxylon* is within the range of generic diagnosis of *Paraphyllanthoxylon* Bailey. In our opinion due to the homogeneity in wood characters, it is difficult to separate *Bridelia* from other genera of the Euphorbiaceae.

### Disjunct distribution pattern and possible migratory path of *Bridelia* from India to Africa-Madagascar and Southeast Asia

The disjunct phytogeography of *Bridelia* with equal distribution of species in Africa and tropical Asia and endemism in the African-Madagascar species are interesting. Based on the molecular data, Li et al. [8] inferred that *Bridelia* separated from its sister genus *Cleistanthus* at 33±5 Ma and suggested that the genus evolved in Southeast Asia and later migrated westward to India and Africa and eastward to Australia. However, this hypothesis didn’t get support from the fossil records of the genus. The present fossil record from the late Oligocene sediments of northeast India is important because during the late Oligocene, suturing of the Indian plate with the Eurasian plate was not complete to facilitate the plant migration between India and Southeast Asia (Figure 3) [11]–[13]. In the light of the oldest fossil evidence of *Bridelia* from northeast India we suggest that the genus evolved most likely during the late Oligocene in northeast India, while the speciation must have occurred during the Miocene followed by the dispersal of the genus from India to Southeast Asia via Myanmar as the suturing of both the aforesaid plates completed during the early Miocene [48], [49]. Our fossil shows affinity with three species of *Bridelia*, namely *B. ovata*, *B. retusa* and *B. stipularis*; this again suggests that the speciation must have occurred after the late Oligocene and most likely during the Miocene as suggested by the molecular data [8] and supported by the diversity in fossil records from the Siwalik of Nepal and India [38], [40]. The climatic conditions also favoured in the evolution of *Bridelia* because the late Oligocene was the time of last significant globally warm climate during the Cenozoic [50] under which the genus evolved and the speciation of the genus in Asia most likely to have occurred during the Middle Miocene Climatic Optimum (MMCO) [50]. All the above facts indicate that *Bridelia* evolved in India during the late Oligocene and after the complete suturing of Indian and Eurasian plates the genus migrated to Southeast Asia via Myanmar and then to Australia, along with several other plant taxa such as *Alphonsea* of the family Annonaceae [20], *Mangifera* [51] and *Semecarpus* of the family Anacardiaceae [18] (Figure 1). Similarly, the stem lineage of *Bridelia* also migrated to Africa via “Iranian Route” [52] during the late Miocene to early Pleistocene and this can be explained on the basis of plate tectonic model. Africa was isolated from Eurasia during the mid-Cretaceous to early Miocene [53]. By the early Miocene, Africa made land connections with east Eurasia via “Iranian Route” (Iranian and Arabian block) [52], and *Bridelia* most likely to have migrated through this route during the Miocene (Figure 1). After reaching to Africa, the stem lineage of the genus speciosed locally due to the availability of free niche and less competition which resulted in the local endemism of the genus in Africa. The speciation of stem lineage of *Bridelia* in Africa again coincides with the climatic condition in Africa i.e. occurrence of aridity in Africa during the late Neogene [54]–[56]. The aforesaid view also gets supports from the molecular phylogenetic study which suggests that the African clade speciosed at *ca* 3±1 Ma [8].

The corridor via “Iranian Route” was not only for *Bridelia* but also common for faunal exchange [52], [57] and several other African plant taxa reported from the Pliocene of western India [58]; this suggests that the migration was in between Africa and east Eurasia.

The migration of *Bridelia* from India to Africa and Southeast Asia again supports the “Out of India” hypothesis [59].

### Palaeofloristics and Palaeoclimate of the Makum Coalfield, Assam

The Makum Coalfield is important in view of its high diversity of plant fossils. The late Oligocene was the time of last significant globally warm period during the Cenozoic and the fossil locality was situated at 10°–15° N palaeolatitude [10]. The known floristic diversity indicates that the family Fabaceae was the most dominant followed by Anacardiaceae, Clusiaceae, Combretaceae, Arecaceae, Annonaceae, Lauraceae and Sapindaceae etc. Most of the aforesaid families have pantropical distribution, while the abundance of palms indicates high water availability.

The families like Annonaceae, Burseraceae, Clusiaceae, Combretaceae, Lecythidaceae, Myristicaceae and Rhizophoraceae are typical pantropical megatherm families [60] whose presence in the palaeoflora provides an evidence that the CMT (mean temperature of the coldest month) was at least not less than 18°C [25]. Similarly, the presence of most dominant family Fabaceae [17] whose abundance and richness covary with the temperature [61], indicates warm climate. The occurrence of families Avicenniaceae and Rhizophoraceae is also very significant in terms of the depositional environment. These families are highly indicative of deltaic, mangrove or lacustrine deposition of coal seams and associated sediments in the Makum Coalfield [15]. The abundance of palms like *Nypa* [23] provides clear evidence of a coastal plain environment where both temperature and humidity remain high throughout the year [62]. Quantitative palaeoclimate reconstruction based on CLAMP analysis on the Makum Coalfield palaeoflora was made by Srivastava et al. [25]. They have used 80 different leaf morphotypes, from the Tirap colliery of the Makum Coalfield, which were analysed by following standard protocol of CLAMP analysis [63]. The CLAMP analysis indicates mean annual temperature (MAT) 28.3±3.7°C, warm month mean temperature (WMMT) 34.2±5.2°C and cold month mean temperature (CMMT) 23.6±5.5°C. The precipitation estimates suggest a marked seasonality in the rainfall pattern showing a wet season with 20 times the rainfall of the dry season. The similar pattern can be seen in Sunderbans lying in the modern Ganges/Brahmaputra/Meghna delta. Therefore, it is suggested that the South Asian Monsoon was already established by the late Oligocene time at an intensity similar to that of today [25].

## Conclusions

Fossil evidences, along with the molecular data are important in studying the evolution and speciation of an organism. In the present paper we have reported fossil leaf of *Bridelia* from the late Oligocene sediments of northeast India and suggested its affinity with *B. ovata*, *B. retusa* and *B. stipularis*. Our fossil data, along with the known fossil records of *Bridelia* from the Neogene sediments of Nepal and India suggests that the genus evolved during the late Oligocene and migration and speciation occurred from India to Southeast Asia via Myanmar and from India to Africa via “Iranian Route” during the Miocene. The present finding fits well with the molecular phylogenetic analysis and plate tectonic models.
